# Ticagrelor to Reduce Myocardial Injury in Patients With High-Risk Coronary Artery Plaque

**DOI:** 10.1016/j.jcmg.2019.05.023

**Published:** 2020-07

**Authors:** Alastair J. Moss, Marc R. Dweck, Mhairi K. Doris, Jack P.M. Andrews, Rong Bing, Rachael O. Forsythe, Timothy R. Cartlidge, Tania A. Pawade, Marwa Daghem, Jennifer B. Raftis, Michelle C. Williams, Edwin J.R. van Beek, Laura Forsyth, Steff C. Lewis, Robert J. Lee, Anoop S.V. Shah, Nicholas L. Mills, David E. Newby, Philip D. Adamson

**Affiliations:** a British Heart Foundation Centre for Cardiovascular Science, University of Edinburgh, Edinburgh, United Kingdom; b Medical Research Council Centre for Inflammation Research, University of Edinburgh, Edinburgh, United Kingdom; c Edinburgh Imaging, Queen’s Medical Research Institute, University of Edinburgh, Edinburgh, United Kingdom; d Edinburgh Clinical Trials Unit, University of Edinburgh, Edinburgh, United Kingdom; e Usher Institute of Population Health Sciences and Informatics, University of Edinburgh, Edinburgh, United Kingdom; f Christchurch Heart Institute, University of Otago, Christchurch, New Zealand

**Keywords:** ^18^F-fluoride, myocardial infarction, troponin, ADP, adenosine diphosphate, CI, confidence interval, CTA, computed tomography angiography, ECG, electrocardiogram, PE, phycoerythrin, PET, positron emission tomography, TBR, tissue to background ratio

## Abstract

**Objectives:**

The goal of this study was to determine whether ticagrelor reduces high-sensitivity troponin I concentrations in patients with established coronary artery disease and high-risk coronary plaque.

**Background:**

High-risk coronary atherosclerotic plaque is associated with higher plasma troponin concentrations suggesting ongoing myocardial injury that may be a target for dual antiplatelet therapy.

**Methods:**

In a randomized, double-blind, placebo-controlled trial, patients with multivessel coronary artery disease underwent coronary ^18^F-fluoride positron emission tomography/coronary computed tomography scanning and measurement of high-sensitivity cardiac troponin I. Patients were randomized (1:1) to receive ticagrelor 90 mg twice daily or matched placebo. The primary endpoint was troponin I concentration at 30 days in patients with increased coronary ^18^F-fluoride uptake.

**Results:**

In total, 202 patients were randomized to treatment, and 191 met the pre-specified criteria for inclusion in the primary analysis. In patients with increased coronary ^18^F-fluoride uptake (120 of 191), there was no evidence that ticagrelor had an effect on plasma troponin concentrations at 30 days (ratio of geometric means for ticagrelor vs. placebo: 1.11; 95% confidence interval: 0.90 to 1.36; p = 0.32). Over 1 year, ticagrelor had no effect on troponin concentrations in patients with increased coronary ^18^F-fluoride uptake (ratio of geometric means: 0.86; 95% confidence interval: 0.63 to 1.17; p = 0.33).

**Conclusions:**

Dual antiplatelet therapy with ticagrelor did not reduce plasma troponin concentrations in patients with high-risk coronary plaque, suggesting that subclinical plaque thrombosis does not contribute to ongoing myocardial injury in this setting. (Dual Antiplatelet Therapy to Reduce Myocardial Injury [DIAMOND]; NCT02110303)

Coronary plaque rupture is the most common cause of acute coronary thrombosis and myocardial infarction (1). Patients who have an increased risk of recurrent plaque rupture events may benefit from intensification of secondary prevention therapy (2). In this regard, the addition of a P2Y_12_ receptor antagonist to low-dose aspirin reduces the risk of cardiovascular death, myocardial infarction, and stroke in patients with recent (3) or previous (4) myocardial infarction. Ticagrelor is an oral, reversible antagonist of the platelet adenosine diphosphate P2Y_12_ receptor. It provides faster, more potent, and more consistent P2Y_12_ inhibition than clopidogrel (5). In the PLATO (Platelet Inhibition and Patients Outcomes) trial of 18,624 patients presenting with acute coronary syndrome, ticagrelor was superior to clopidogrel for the prevention of cardiovascular events and death (3). Moreover, the prolonged use of dual antiplatelet therapy after myocardial infarction continues to reduce cardiovascular events, albeit at the expense of increased rates of major bleeding (4). Thus, there is a clinical need to improve the risk stratification of patients to enable physicians to better select “vulnerable” patients who may benefit from extended duration of dual antiplatelet therapy.

A novel approach for assessing patients at high risk of coronary plaque rupture is using positron emission tomography (PET) and coronary computed tomography angiography (CTA). This technique uses the radiotracer ^18^F-fluoride to identify regions of increased disease activity in coronary artery plaques. Previous studies have shown that coronary ^18^F-fluoride uptake correlates with a high-risk cardiovascular profile and identifies ruptured coronary plaques in patients with recent myocardial infarction 6, 7. Importantly, we have previously reported an association between increased coronary ^18^F-fluoride uptake and higher plasma high-sensitivity cardiac troponin I concentrations in patients with stable coronary artery disease (7). Silent plaque rupture is common, and subclinical plaque thrombus formation is a frequent incidental post-mortem finding in patients with multivessel coronary artery disease who have died of noncardiovascular causes (8). This result suggests that coronary ^18^F-fluoride uptake may identify high-risk plaque that is associated with thrombus formation and subclinical myocardial injury from microemboli. If correct, this would potentially be modifiable with intensive dual antiplatelet therapy.

The current study assessed whether coronary ^18^F-fluoride activity identifies patients with stable multivessel coronary artery disease who respond favorably to ticagrelor as assessed by a reduction in high-sensitivity cardiac troponin I concentrations.

## Methods

### Study design

This investigator-initiated, double-blind, randomized, parallel-group, placebo-controlled trial was conducted at a single center in Edinburgh, United Kingdom. The study was approved by the local institutional review board, the Scottish Research Ethics Committee (REC reference: 14/SS/0089), the Medicines and Healthcare products Regulatory Agency, and the United Kingdom Administration of Radiation Substances Advisory Committee. It was performed in accordance with the Declaration of Helsinki. All patients provided written informed consent before any study procedures were initiated.

### Study population

Patients were recruited between March 2015 and March 2017. Patients were included if they were ≥40 years of age and already receiving aspirin therapy with angiographically proven multivessel coronary artery disease, defined as at least 2 major epicardial vessels with any combination of either: 1) >50% luminal stenosis; or 2) previous revascularization (percutaneous coronary intervention or coronary artery bypass graft surgery). Patients were excluded if they had any of the following criteria: an acute coronary syndrome within the last 12 months, any ongoing indication for dual antiplatelet therapy, concurrent thienopyridine (clopidogrel or prasugrel) or oral anticoagulant therapy, or percutaneous coronary intervention or coronary artery bypass graft surgery within the last 3 months. Full eligibility criteria are provided in Supplemental Table 1.

### Trial intervention and randomization

Patients were randomly assigned 1:1 to receive either ticagrelor 90 mg twice daily or matched placebo tablets (AstraZeneca, Cambridge, United Kingdom). Randomization was performed by using a Web-based system that ensured allocation concealment, with treatment allocation incorporating minimization based on age (<65 and ≥65 years of age), sex, baseline plasma high-sensitivity troponin I concentration (≤5.1 and >5.1 ng/l), and the presence or absence of coronary ^18^F-fluoride uptake. A random element was included with a 1 in 10 chance of the determined treatment allocation being switched to the other treatment arm.

### Study procedures

All patients underwent a baseline assessment to confirm eligibility and measurement of plasma high-sensitivity cardiac troponin I concentration and platelet–monocyte aggregates. An electrocardiogram (ECG)-gated ^18^F-fluoride PET/coronary CTA was performed after patients had received 50 to 100 mg of oral metoprolol if their resting heart rate was >65 beats/min before the intravenous administration of 250 MBq of ^18^F-fluoride. After 60 min, patients were imaged with a hybrid PET/CT scanner (64-multidetector Biograph mCT, Siemens Medical Systems, Erlangen, Germany). Attenuation correction CT scans were performed before the acquisition of ECG-gated list-mode PET data using a single 30-min bed position centered on the heart. Finally, an ECG-gated coronary CTA was performed in mid-diastole during held expiration after administration of sublingual glyceryl trinitrate.

### Image analysis

PET images were reconstructed in diastole (50% to 75% of the R-R interval, 2 iterations, 21 subsets; Siemens Ultra-HD algorithm) and fused with contrast-enhanced coronary CTA. Analysis of the CT images was performed by using dedicated software (Vitrea Advanced, Toshiba Medical Systems, Otawara, Tochigi Prefecture, Japan) with multiplanar reformatting for plaque analysis as required. Coronary arteries with a diameter ≥2 mm were assessed according to the 18-segment Society of Cardiovascular Computed Tomography model. Qualitative and semiquantitative analysis of the PET images was performed by trained observers using an OsiriX workstation (OsiriX version 3.5.1, 64-bit, OsiriX Imaging Software, Geneva, Switzerland).

The analysis of coronary ^18^F-fluoride activity has been previously described 6, 7. In brief, visual assessment for increased coronary ^18^F-fluoride activity was performed on both a per-patient level and a per-segment basis. For a signal to be co-localized to the coronary artery, an atherosclerotic plaque had to be present on the coronary CTA image, and the increased pattern of radiotracer had to arise from the coronary artery and follow its course over >5 mm in 3 dimensions on orthogonal views. Semi-quantitative PET analysis was undertaken for all proximal coronary segments in addition to any atherosclerotic segment with focal ^18^F-fluoride activity as described earlier. Maximum standardized uptake values were measured within regions of interest. Correction was made for uptake in a referent proximal coronary plaque with no evidence of increased ^18^F-fluoride activity. To calculate coronary target to background ratios (TBRs), coronary maximum standardized uptake values were divided by these background measures, providing TBR_MAX_. Coronary ^18^F-fluoride activity with TBR_MAX_ >1.25 was classified a high-risk plaque.

### High-sensitivity cardiac troponin I

Plasma high-sensitivity cardiac troponin I concentrations were measured by using the *ARCHITECT* STAT assay (Abbott Laboratories, Abbott Park, Illinois). The limit of detection is 1.0 ng/l with an interassay coefficient of variation <10% at 4.7 ng/l (9). The upper reference limit (99th centile) based on 4,590 samples from healthy men and women is 34 ng/l for men and 16 ng/l for women (10). Samples were collected at baseline, 30 days, and 3, 6, 9, and 12 months. A value of 0.5 ng/l was imputed for troponin values below the limit of detection.

### Platelet function analysis

Platelet and monocyte activation in response to adenosine diphosphate (ADP) was determined according to flow cytometry, as previously described (11). These analyses were performed by a single technician blinded to study allocation with the results of these investigations withheld from the study team until after trial database lock. Briefly, peripheral venous blood was obtained from all participants at the baseline and 1-month visits. Blood was drawn by clean venipuncture of a large antecubital vein using a 19-gauge needle, and care was taken to ensure a smooth blood draw without venous stasis. Blood was collected into tubes containing a direct thrombin inhibitor, D-phenylalanine-L-prolyl-L-arginine chloromethyl ketone (Cambridge Biosciences, Cambridge, United Kingdom). Tubes were gently inverted to ensure mixing of whole blood with anticoagulant.

Immunolabeling and flow cytometry were performed in whole blood to avoid centrifugation and washing steps, which can lead to artifactual platelet activation. All chemicals were obtained from BD Biosciences (Oxford, United Kingdom). Aliquots of whole blood (50 μl) were incubated with anti–CD14-Allophycocyanin, anti–CD42a-fluorescein isothiocyanate, anti–CD11b-PE-Cyanine7, anti–CD62p-phycoerythrin (PE), and isotype-matched controls for 20 min at room temperature in Eppendorf tubes (Eppendorf, Hamburg, Germany) with and without ADP (at a final concentration of 20 μmol/l). Thereafter, samples were fixed with 1% paraformaldehyde (P-selectin) or FACS Lysing (Becton Dickinson) (platelet–monocyte aggregates). All samples were analyzed within 24 h by using a FACSCalibur flow cytometer (Becton Dickinson, Franklin Lakes, New Jersey). Data analysis was performed by using FlowJo v10 (Treestar, Woodburn, Oregon). A medium flow setting was used to minimize leukocyte–platelet coincident events. Monocytes were identified based on their forward and side scatter characteristics and then by triggering on FL-4 to identify CD14-PE–positive monocytes and exclude large granular lymphocytes. For each measurement, a minimum of 2,500 monocytes were collected. Platelet–monocyte aggregates were defined as monocytes positive for CD42a. All results are expressed as geometric mean of fluorescence. P-selectin expression was defined as CD42a-fluorescein isothiocyanate–positive platelets that were also positive for CD62p-PE.

### Study endpoints

The pre-specified primary endpoint was high-sensitivity cardiac troponin I concentrations at 30 days in patients with increased coronary ^18^F-fluoride activity. Secondary endpoints were plasma high-sensitivity cardiac troponin I concentration at 30 days in patients without coronary ^18^F-fluoride activity, and plasma high-sensitivity troponin I concentration over 1 year. Adverse events were recorded in all patients who received a single dose of study medication and included bleeding events categorized according to PLATO criteria as major life-threatening, other major, minor, or minimal bleeding (3).

### Sample size

In patients with increased coronary ^18^F-fluoride uptake, we previously reported that mean ± SD troponin concentrations were more than double those in patients without increased coronary ^18^F-fluoride uptake (7.9 ± 9.3 ng/l vs. 3.1 ± 1.9 ng/l; p = 0.047) (7). It was estimated that ticagrelor would reduce the troponin concentration by one-half. Forty-eight patients per treatment arm were required to achieve 80% power at 2-sided p < 0.05. After allowing for 15% dropout, we estimated that 55 patients will be required per treatment arm. Previous studies had found that 45% of patients with advanced but stable coronary artery disease exhibited increased coronary ^18^F-fluoride uptake; thus, a total sample size of 250 patients was estimated to be required to identify 110 patients with increased coronary ^18^F-fluoride activity. Termination of further recruitment could be authorized by the trial steering committee once a per-protocol population of 110 patients with increased coronary ^18^F-fluoride activity had been randomized to treatment and completed the primary endpoint at 30 days.

### Statistical analysis

Categorical data are presented by using counts and proportions, and continuous variables are presented by using mean ± SD, median (interquartile range), minimum, maximum, and number of patients. Participants were removed from formal statistical analysis where data were missing for that outcome variable. All analyses (except safety) were performed on a per-protocol population that excluded participants without a blood sample, or whose compliance was <80% for the study medication, at the 30-day visit. For the primary analysis, the change in troponin I concentration from baseline to 30 days was compared between the 2 treatment groups (ticagrelor and placebo) by using linear regression, adjusting for the minimization variables in patients with increased coronary ^18^F-fluoride uptake. Before analysis, tests for normality were undertaken and, where data were skewed, logarithmic transformation was performed. Central estimates and 95% confidence intervals (CIs) were calculated. Similar analyses were performed for secondary outcomes.

In post hoc testing, we compared baseline troponin concentrations between patients with and without evidence of coronary ^18^F-fluoride activity and also confirmed treatment efficacy by comparison of ADP-stimulated platelet activation between the 2 trial intervention groups (ticagrelor vs. placebo). For 1-year evaluation of changes in cardiac troponin I concentrations, an adjusted linear regression model (adjusted for the minimization variables) was generated and descriptive statistics were presented for the AUC. For missing values, the value was imputed linearly from adjacent measurements. Adjustment for age was performed as a linear term. To determine whether there was efficacy of ticagrelor using a baseline troponin I concentration ≥5 ng/l, a post hoc comparison was made between groups using the method described in the primary analysis. For all analyses, a 2-sided p value <0.05 was taken as statistically significant. Statistical analysis was performed by using SAS version 9.4 (SAS Institute, Inc., Cary, North Carolina) with the primary analysis validated by a second statistician in the Edinburgh Clinical Trials Unit. Post hoc analyses were performed separately from the primary statistical analysis plan using R version 3.4.0 (R Foundation for Statistical Computing, Vienna, Austria) by 1 of the authors (P.D.A.).

## Results

### Study population

**Figure 1:**
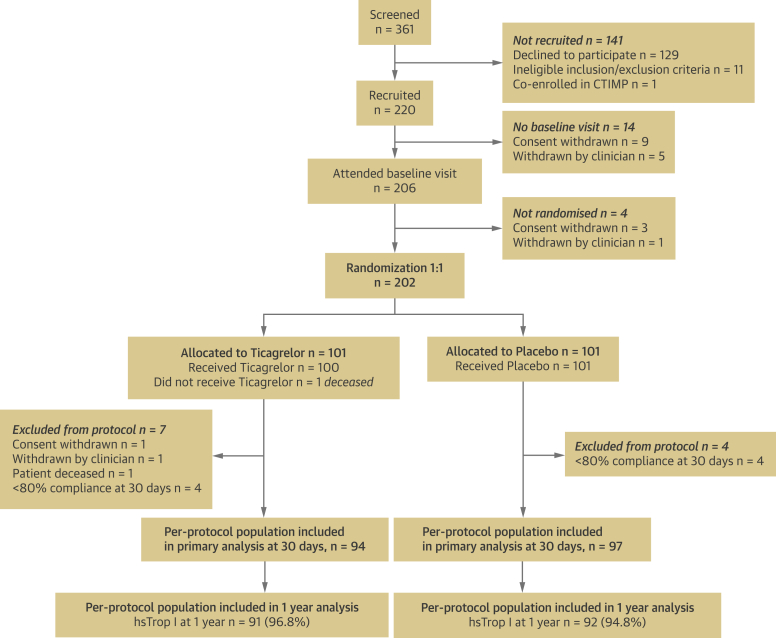
CONSORT Diagram Flow diagram of the progress through the phases of the randomized trial between ticagrelor and placebo groups. CTIMP = Clinical Trial of an Investigational Medicinal Product; hsTrop I = high-sensitivity troponin I.

**Figure 2:**
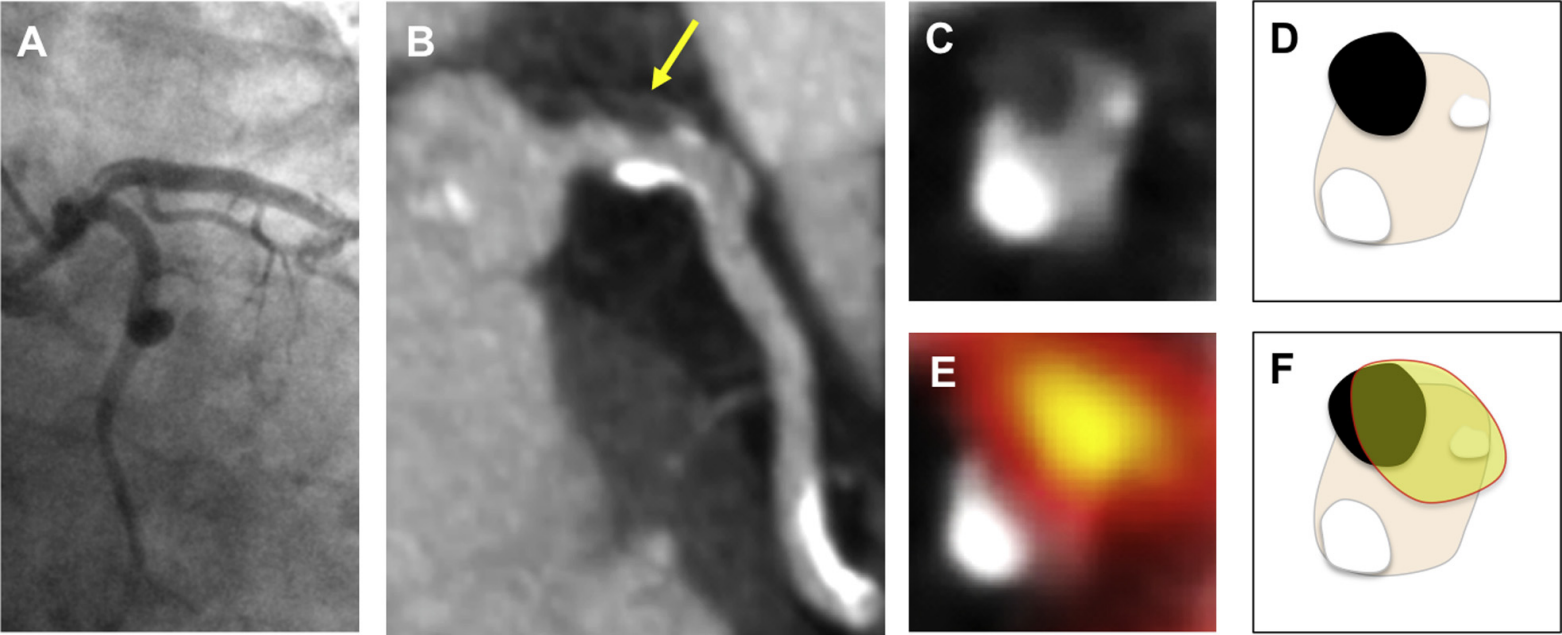
Intracoronary Thrombus and Coronary ^18^F-Fluoride Activity **(A and B)** A 72-year-old woman with intracoronary thrombus in the left main stem **(arrow)**. (**C and D**, schematic) Axial reconstructions show a nonobstructive intracoronary thrombus at the 11 o’clock position with coronary calcification at the 2 o’clock and 7 o’clock positions. (**E and F**, schematic). ^18^F-fluoride activity was present in the coronary plaque.

A total of 361 patients were screened and 202 patients were randomized to treatment after baseline coronary ^18^F-fluoride PET/coronary CTA imaging (Figure 1). Eleven patients discontinued the study early due to withdrawal of consent (n = 1), new diagnosis of malignancy on baseline PET/coronary CTA (n = 1), <80% compliance with study medication at 30 days (n = 8), and sudden unexpected death before receiving study medication (n = 1). The randomized groups were well matched for the presence of cardiovascular risk factors and represented a high-risk cohort, with 70% having a history of acute coronary syndrome (median 2.25 years before study enrollment) (Table 1). A per-protocol population of 191 patients (mean age: 65.9 ± 8.3 years; 80% male) had both blood sampling at 30 days and ≥80% compliance with the study medication, comprising 94 patients in the ticagrelor group and 97 patients in the placebo group. A total of 120 (62.8%) patients had evidence of coronary ^18^F-fluoride activity in at least 1 epicardial vessel (Table 2, Figure 2).

**Table 1:** Baseline Characteristics of the Study Population

	Total Randomized Population (N = 202)	Per-Protocol Population (n = 191)	Ticagrelor (n = 94)	Placebo (n = 97)	p Value (Ticagrelor vs. Placebo)∗
Age, yrs	65.9 ± 8.2	65.9 ± 8.3	65.5 ± 8.4	66.3 ± 8.1	0.504
Male	162 (80)	152 (80)	74 (79)	78 (80)	0.912
Body mass index, kg/m^2^	29.8 ± 5.2	29.7 ± 5.0	30.0 ± 5.2	29.4 ± 4.9	0.413
Medical history					
History of acute coronary syndrome	143 (71)	134 (70)	65 (69)	69 (71)	0.887
Days between ACS and randomization	821 (620–1,056)	821 (625–1,037)	800 (620–970)	861 (646–1,081)	
Percutaneous coronary intervention	163 (81)	154 (81)	75 (80)	79 (81)	0.915
Coronary artery bypass grafting	40 (20)	38 (20)	18 (19)	20 (21)	0.942
Hypertension	113 (56)	105 (55)	52 (55)	53 (55)	1.000
Hypercholesterolemia	195 (97)	185 (97)	93 (99)	92 (95)	0.228
Diabetes mellitus	39 (19)	36 (19)	19 (20)	17 (18)	0.772
Previous stroke/transient ischemic attack	4 (2)	4 (2)	2 (2)	2 (2)	1.000
History of atrial fibrillation	5 (2)	5 (3)	4 (4)	1 (1)	0.346
Peripheral vascular disease	8 (4)	7 (4)	1 (1)	6 (6)	0.134
Medications					
Aspirin	202 (100)	191 (100)	94 (100)	97 (100)	NA
Statin	192 (95)	182 (95)	92 (98)	90 (93)	0.188
Beta-blocker	138 (68)	130 (68)	66 (70)	64 (66)	0.637
Angiotensin-converting enzyme inhibitor/angiotensin II receptor blocker	155 (77)	145 (76)	68 (72)	77 (79)	0.333
Hemoglobin, g/dl	14.0 ± 1.3	14.0 ± 1.3	14.2 ± 1.2	13.8 ± 1.3	0.034
Estimated glomerular filtration rate, ml/min/1.73 m^2^					0.547
31–60	23 (11)	22 (12)	9 (10)	13 (13)	
>60	179 (89)	169 (88)	85 (90)	84 (87)	
Total cholesterol, mg/dl	162 ± 39	162 ± 39	162 ± 39	162 ± 35	0.852
High-density lipoprotein, mg/dl	46 ± 12	46 ± 12	43 ± 15	46 ± 12	0.128
Low-density lipoprotein, mg/dl	89 ± 31	89 ± 31	85 ± 35	89 ± 27	0.377
Triglycerides, mg/dl	159 ± 97	151 ± 97	159 ± 106	151 ± 80	0.556

Values are mean ± SD, n (%), or median (interquartile range).

ACS = acute coronary syndromes.

∗ Post-hoc analysis.

**Table 2:** Plasma High-Sensitivity Cardiac Troponin I Concentration in the Per-Protocol Population

	Overall (N = 191)	Ticagrelor (n = 94)	Placebo (n = 97)	p Value∗
Coronary ^18^F-fluoride uptake, ng/l				
N	120	59	61	
Baseline	3.8 ± 2.9	4.2 ± 2.9	3.5 ± 3.0	0.197
30 days	3.6 ± 2.7	4.1 ± 2.5	3.2 ± 2.9	0.072
Ratio of 30 days to baseline	0.95 ± 1.87	0.97 ± 2.13	0.93 ± 1.59	0.907
No coronary ^18^F-fluoride uptake, ng/l				
N	71	35	36	
Baseline	2.5 ± 2.6	2.5 ± 2.8	2.4 ± 2.4	0.872
30 days	2.4 ± 2.7	2.4 ± 2.8	2.3 ± 2.6	0.877
Ratio of 30 days to baseline	0.97 ± 1.68	0.97 ± 1.77	0.96 ± 1.59	

Values are geometric mean ± geometric SD, back-transformed from log-transformed values, unless otherwise indicated.

∗ Post-hoc analysis.

The geometric mean troponin I concentration at baseline was 3.8 (geometric SD: 2.9) ng/l in patients with increased coronary ^18^F-fluoride activity compared with 2.5 (geometric SD: 2.6) ng/l in those without uptake (p = 0.004) (Table 2) from a post hoc analysis.

### Effect of ticagrelor on platelet function

**Figure 3:**
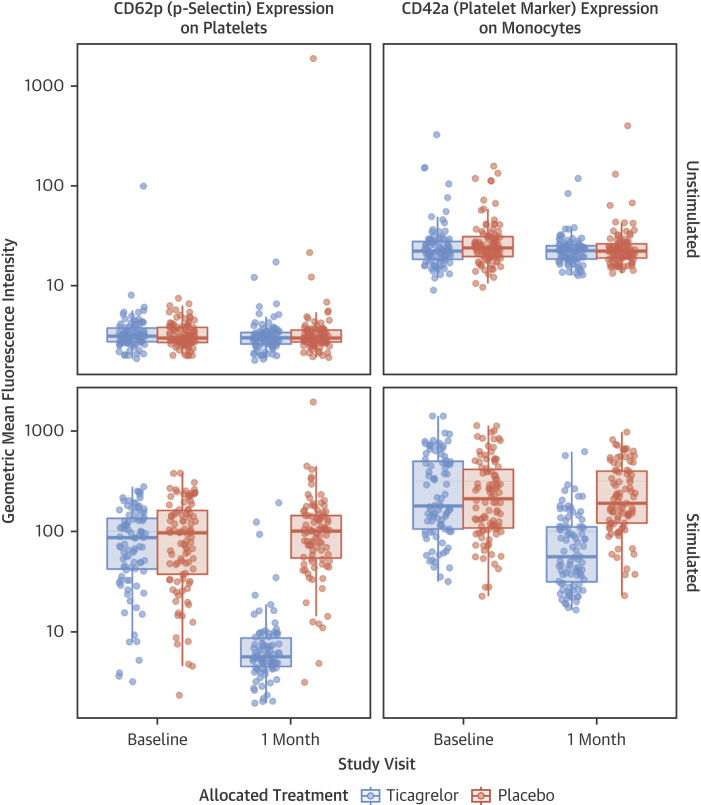
Flow Cytometry Assessment of Platelet Activation at Baseline and 30 Days Unstimulated **(upper panels)** and adenosine diphosphate (20 μmol/l)-stimulated **(lower panels)** levels of platelet activation (P-selectin expression) and platelet–monocyte aggregates.

Baseline platelet and monocyte reactivities were well balanced between treatment arms. Consistent with its known pharmacological action, ticagrelor markedly inhibited platelet P-selectin expression and reduced the formation of platelet–monocyte aggregates after ex vivo stimulation with ADP (all, p < 0.001) (Figure 3). Ticagrelor had no effect on 30-day unstimulated platelet activation (all, p > 0.05). These results were derived from post hoc analysis.

### Effect of ticagrelor on high-sensitivity troponin I at 30 days

For the primary endpoint, there was no effect of ticagrelor on troponin I at 30 days in patients who had increased coronary ^18^F-fluoride activity (ratio of geometric means ticagrelor vs. placebo: 1.11; 95% CI: 0.90 to 1.36; p = 0.32) (Table 3). Similarly, among the 71 (37.2%) patients with no discernible coronary ^18^F-fluoride activity, there was no difference in the 30-day troponin I concentration between ticagrelor and placebo (ratio of geometric means: 1.02; 95% CI: 0.80 to 1.31; p = 0.87).

**Table 3:** Plasma High-Sensitivity Cardiac Troponin I Concentration at 30 Days for the Per-Protocol Population

	Adjusted Geometric Mean (GSE)		Ratio of Geometric Means (95% CI)	p Value
	Ticagrelor	Placebo		
Cardiac troponin I, ng/l (^18^F-fluoride activity)	3.8 (1.1)	3.4 (1.1)	1.11 (0.90 to 1.36)	0.32
Cardiac troponin I, ng/l (no ^18^F-fluoride activity)	2.4 (1.1)	2.3 (1.1)	1.02 (0.80 to 1.31)	0.87

Estimates are back-transformed estimates from analysis of log-transformed values at 30 days adjusting for age, sex, and log-transformed baseline troponin. Ratio of geometric means is ticagrelor divided by placebo.

CI = confidence interval; GSE = geometric standard error.

**Figure 4:**
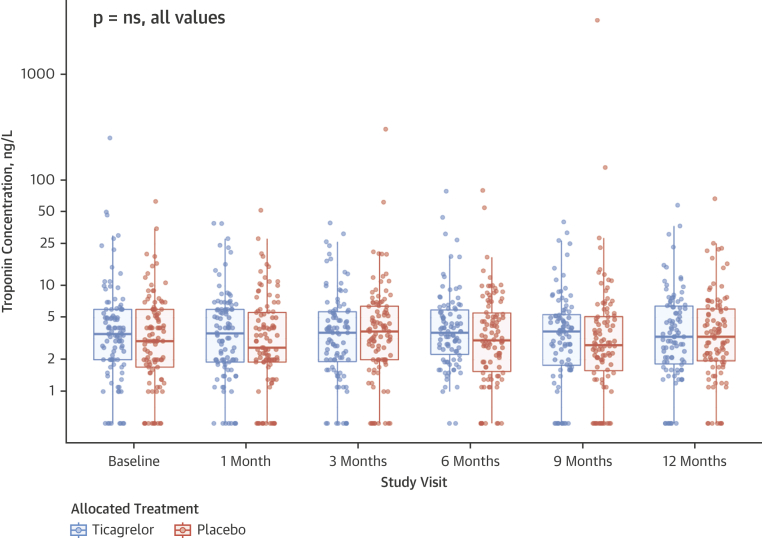
Plasma High-Sensitivity Cardiac Troponin I Concentration Over 1 Year Box-whisker plot of individual patient-level plasma high-sensitivity troponin I concentration in ticagrelor **(blue)** and placebo **(red)** groups at baseline and 1, 3, 6, 9, and 12 months. Median and interquartile range are given for each time point.

We explored whether a reduction in cardiac troponin I could be shown over 12 months. Twelve-month troponin I concentrations were measured in 183 (95.8%) patients, comprising 91 (96.8%) patients in the ticagrelor group and 92 (94.8%) patients in the placebo group. There was no difference in area under the concentration curve of troponin I over 12 months between the ticagrelor and placebo groups (ratio of geometric means: 0.92; 95% CI: 0.74 to 1.13; p = 0.42) (Figure 4, Table 4). Post hoc analysis of the subset of patients with a baseline troponin I concentration ≥5 ng/l (ticagrelor: n = 34, baseline geometric mean = 10.3 ng/l; placebo: n = 33, baseline geometric mean = 8.7 ng/l) found no change in troponin I concentration at 30 days (p = 0.89) or 12 months (p = 0.86) (Supplemental Figure 1, Supplemental Table 2).

**Table 4:** Plasma High-Sensitivity Cardiac Troponin I Concentration Over 1 Year for the Per-Protocol Population

	Adjusted Geometric Mean (GSE)		Ratio of Geometric Means (95% CI)	p Value
	Ticagrelor	Placebo		
AUC from 30 days to 1 yr (^18^F-fluoride activity)	3.7 (1.1)	4.4 (1.1)	0.86 (0.63 to 1.17)	0.33
AUC from 30 days to 1 yr (no ^18^F-fluoride activity)	2.4 (1.1)	2.3 (1.1)	1.04 (0.84 to 1.28)	0.70

Estimates are back-transformed estimates from analysis of log-transformed values area under curve (AUC) from 30 days to 1 year adjusting for age, sex, and log-transformed baseline troponin. Ratio of geometric means is ticagrelor divided by placebo.

Abbreviations as in Table 3.

### Safety outcomes

There were no suspected unexpected serious adverse reactions over the course of the study. Serious adverse events occurred in 7 (7%) of 100 patients who received at least 1 single dose of ticagrelor and 15 (11.9%) of 101 patients who were administered placebo (Supplemental Table 3). There were no reported major life-threatening or other major bleeding events over the course of the study. Minimal bleeding events (bruising) were reported in 64 (64.0%) patients in the ticagrelor group and 12 (11.9%) patients in the placebo group (Supplemental Table 4). Dyspnea episodes occurred in 24 (24%) patients in the ticagrelor group compared with 8 (7.9%) patients in the placebo group at 1 year.

## Discussion

In this randomized, placebo-controlled trial, we found no evidence that ticagrelor 90 mg twice daily reduces plasma high-sensitivity cardiac troponin I concentrations in patients with high-risk plaque and established multivessel coronary artery disease (Central Illustration). This outcome suggests that, in patients with high-risk coronary plaque, plasma cardiac troponin I concentrations are not attributable to subclinical myocardial injury from thrombotic microembolic injury.

**Central Illustration:**
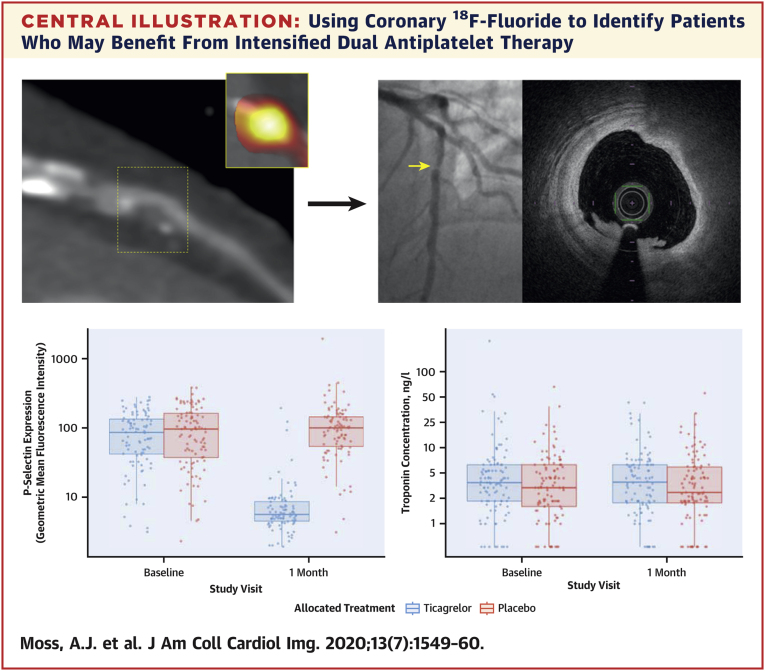
Using Coronary ^18^F-Fluoride to Identify Patients Who May Benefit From Intensified Dual Antiplatelet Therapy Coronary ^18^F-fluoride positron emission tomography was used to identify high-risk coronary plaque in patients with stable multivessel coronary artery disease. Randomization to intensified dual antiplatelet therapy with ticagrelor did not reduce plasma high-sensitivity cardiac troponin I concentrations at 30 days in patients with high-risk plaque.

The current study has several important strengths. This trial is the first to use PET/coronary CTA imaging with ^18^F-fluoride to identify patients with high-risk coronary plaque who may be at heightened risk of future coronary events and thereby have the most to gain from potent dual antiplatelet therapies. It is also the largest trial to date using coronary plaque PET imaging. Although previous PET studies have used ^18^F-fluorodeoxyglucose to visualize inflammation within the carotid arteries as a surrogate to guide intensification of atherosclerotic therapy 12, 13, the coronary and cerebral vascular beds differ both with respect to their underlying molecular pathophysiology and also in response to the treatment effect using ticagrelor 3, 14. Second, our unique study design enabled high-risk patients with multivessel coronary disease and in vivo evidence of disease activity to be precisely phenotyped before randomization in a manner that can seldom be achieved in larger clinical outcome trials 3, 15. Finally, this study is the first prospective randomized controlled trial to use high-sensitivity cardiac troponin I concentrations as a surrogate outcome measure for assessing future cardiovascular risk.

In trying to understand why P2Y_12_ inhibition did not reduce cardiac troponin in this study, it is worth addressing some of the underlying assumptions in the trial design. Does coronary ^18^F-fluoride activity identify patients with high-risk plaque? Studies have found that ^18^F-fluoride holds potential in identifying culprit plaques in the coronary circulation by classifying patients who have a high-risk cardiovascular phenotype and culprit plaque rupture after type 1 myocardial infarction 6, 7. Histological validation indicates that ^18^F-fluoride preferentially binds to microcalcification in regions of plaque mineralization, a key component of high-risk plaque (16). Hydroxyapatite, the most common form of atherosclerotic microcalcification, is extruded from apoptotic macrophages and accumulates within necrotic cores, where it may destabilize the structural integrity of the fibrous cap 17, 18. The identification of abnormal material composition of the arterial wall has clinical relevance, as these regions may lead to atherosclerotic plaque rupture manifesting as myocardial infarction, stroke, or aneurysm rupture 7, 19, 20. In our cohort, the frequency of ^18^F-fluoride activity (>60%) in stable coronary artery disease is similar to previous estimates in patients with a high burden of coronary artery disease and previous myocardial infarction (6). This research confirms the high prevalence of coronary ^18^F-fluoride activity in stable patients with multivessel coronary artery disease who had intensification of antiplatelet therapy may be considered.

A key question is whether troponin measurements below the 99th centile reflect subclinical plaque rupture with accompanying distal microvascular embolization, as has previously been posited (21). In this regard, some therapies directed at reducing the risk of atherosclerotic plaque rupture, such as pravastatin, both modify troponin concentrations and reduce the risk of myocardial infarction 22, 23. In contrast, strategies that have failed to show a reduction in cardiovascular events in the context of stable coronary artery disease, such as coronary revascularization, attenuation of plaque inflammation, and inhaled therapies for respiratory disease, have not correlated with a reduction in serial troponin concentration 24, 25, 26. If subclinical plaque thrombosis is the dominant mechanism underlying detectable troponin I concentrations in patients with stable coronary artery disease, a reduction in troponin I concentration would be expected after administration of potent antiplatelet therapy. The lack of response to ticagrelor in this study would suggest that other contributing mechanisms to myocardial injury should be considered. The emergence of newer therapies (e.g., sodium–glucose cotransporter 2 inhibition) that lower blood pressure may reduce troponin concentrations through an improvement in myocardial remodeling, further raising doubts over the subclinical plaque rupture hypothesis 27, 28. In this study, high-sensitivity cardiac troponin I concentrations were higher in patients with ^18^F-fluoride activity, although the differences were small and below the established risk stratification threshold of 5 ng/l 9, 22, 29. It therefore seems unlikely that troponin at these concentrations reflects subclinical plaque rupture, and it is perhaps unsurprising that ticagrelor treatment did not result in an early or late reduction in troponin concentration.

Previous reports have suggested that there is a high incidence of subclinical intracoronary thrombus in patients with apparently stable coronary artery disease. Indeed, some have suggested that this outcome occurs in as many as 1 in 7 patients (8). If this is the case, it would seem that intracoronary thrombus does not track with troponin. This suggests that better noninvasive markers of coronary thrombosis, such as novel PET tracers (30) or noninvasive imaging (31), are needed to use as biomarkers of cardiovascular risk and antithrombotic therapeutic efficacy.

### Study limitations

The current study had a modest sample size to assess the impact of ticagrelor on a readily available plasma biomarker, and a larger study would be required to assess clinical outcomes of ticagrelor use in patients with stable coronary artery disease and coronary ^18^F-fluoride activity. The low baseline troponin I concentrations observed in this study may have limited power to show the benefit of ticagrelor in this population. Enrichment of the population by selecting patients with higher troponin I concentrations before study entry may need to be considered for future trials. It should also be acknowledged that this study was conducted in a single center with expertise in coronary ^18^F-fluoride imaging, and the methods for analyzing coronary ^18^F-fluoride activity are subject to a number of operator- and scan-dependent variables. Although recent reports have suggested that coronary ^18^F-fluoride activity may hold prognostic value in stratifying high-risk populations (32), larger prospective studies evaluating the prognostic utility of coronary ^18^F-fluoride activity in patients with cardiovascular disease are ongoing (NCT02278211).

## Conclusions

In patients with multivessel coronary artery disease and in vivo coronary ^18^F-fluoride activity, we found no evidence that intensification of antiplatelet therapy using ticagrelor 90 mg twice daily reduces plasma high-sensitivity cardiac troponin I concentration at 30 days or 1 year. These findings suggest that in this group of patients, plasma high-sensitivity cardiac troponin I concentrations may not be a suitable marker for predicting efficacy of P2Y_12_ inhibition.

Perspectives **COMPETENCY IN MEDICAL KNOWLEDGE:** High-risk coronary artery plaque and plasma high-sensitivity cardiac troponin I concentrations are associated with increased rates of cardiovascular events.

**COMPETENCY IN PATIENT CARE:** Patients with stable coronary artery disease and an increased risk of cardiovascular events may benefit from extended therapy with P2Y_12_ inhibition.

**TRANSLATIONAL OUTLOOK 1:** Although this study used an early biomarker (30-day plasma high-sensitivity cardiac troponin I concentration) to evaluate drug efficacy, coronary ^18^F-fluoride activity did not seem to be useful in identifying patients who may benefit from extended P2Y_12_ inhibition.

**TRANSLATIONAL OUTLOOK 2:** A detailed phenotype of coronary plaque disease activity using PET is both feasible and practical in the setting of a randomized, placebo-controlled trial.
