# Supplementary material for: Ticagrelor to Reduce Myocardial Injury in Patients With High-Risk Coronary Artery Plaque
Source: JACC Cardiovasc Imaging. 2020 Jul;13(7):1549–60. doi: 10.1016/j.jcmg.2019.05.023 (PMC7342015; doi:10.1016/j.jcmg.2019.05.023)

**Supplemental Table 1.**

Inclusion and Exclusion Criteria

| **Inclusion Criteria** |
| --- |
| For inclusion in the study subjects should fulfil the following criteria:   1. Patients aged ≥40 years with angiographically proven multivessel coronary artery disease defined as at least two major epicardial vessels with any combination of either (a) >50% luminal stenosis, or (b) previous revascularization (percutaneous coronary intervention or coronary artery bypass graft surgery). 2. Provision of informed consent prior to any study specific procedures 3. Receiving aspirin |
| **Exclusion Criteria** |
| Subjects should not enter the study if any of the following exclusion criteria are fulfilled:   1. An acute coronary syndrome within the last 12 months 2. An indication for dual anti-platelet therapy, such as drug eluting stent 3. Receiving thienopyridine therapy such as clopidogrel or prasugrel 4. Percutaneous coronary intervention or coronary artery bypass graft surgery within the last 3 months 5. Inability or unwilling to give informed consent 6. Women who are pregnant, breastfeeding or of child-bearing potential (women who have experienced menarche, are pre-menopausal and have not been sterilised) will not be enrolled into the trial 7. Known hypersensitivity to ticagrelor or one of its excipients 8. Active pathological bleeding or bleeding diathesis 9. Significant thrombocytopenia: platelets <100 x 10^9^ /L 10. History of intracranial hemorrhage 11. Moderate to severe liver impairments (Child’s Grade B or C) 12. Maintenance therapy with strong CYP3A4 inhibitors, such as ketoconazole, nefazodone, ritonavir, indinavir, atazanavir, or clarithromycin 13. Major intercurrent illness of life expectancy <1 year 14. Renal dysfunction (eGFR ≤30 mL/min/1.73m^2^) 15. Contraindication to iodinated contrast agents 16. Planned coronary revascularization or major non-cardiac surgery in the next 12 months 17. Maintenance therapy with simvastatin or lovastatin at doses greater than 40mg daily 18. Receiving oral anticoagulants including warfarin, rivaroxaban, dabigatran or apixaban |

**Supplemental Table 2.**

Post-hoc analysis of efficacy of Ticagrelor in patients with troponin I concentration *≥ 5 ng/L*

| PLACEBO | Baseline 18F-fluoride uptake on PET-CT | | | |
| --- | --- | --- | --- | --- |
|  | Negative | Negative | Positive | Positive |
|  | Baseline hs-cTnI <5 ng/L | Baseline hs-cTnI ≥5 ng/L | Baseline hs-cTnI <5 ng/L | Baseline hs-cTnI ≥5 ng/L |
| Baseline hs-cTnI, geometric mean (95% CI) | 1.8 (1.4 to 2.3) | 7.7 (5.9 to 10.1) | 1.8 (1.4 to 2.3) | 9.3 (7.2 to 12.1) |
| 30 day hs-cTnI, geometric mean (95% CI) | 1.7 (1.3 to 2.3) | 7.1 (4.1 to 12.1) | 1.8 (1.4 to 2.3) | 8.3 (6.1 to 11.2) |

| TICAGRELOR | Baseline 18F-fluoride uptake on PET-CT | | | |
| --- | --- | --- | --- | --- |
|  | Negative | Negative | Positive | Positive |
|  | Baseline hs-cTnI <5 ng/L | Baseline hs-cTnI >5 ng/L | Baseline hs-cTnI <5 ng/L | Baseline hs-cTnI >5 ng/L |
| Baseline hs-cTnI, geometric mean (95% CI) | 1.8 (1.4 to 2.3) | 8.7 (4.7 to 16.3) | 2.3 (1.9 to 2.9) | 10.3 (7.6 to 15.8) |
| 30 day hs-cTnI, geometric mean (95% CI) | 1.7 (1.3 to 2.2) | 10.2 (5.2 to 20.1) | 2.7 (2.1 to 3.4) | 8.3 (6.1 to 11.2) |

**Supplemental Table 3.**

Serious Adverse Events for safety population

|  |  | **Ticagrelor**  **n=100** | | **Placebo**  **n=101** | | **Overall**  **n=201** | |
| --- | --- | --- | --- | --- | --- | --- | --- |
|  |  | **Number of Events** | **Number of Patients** | **Number of Events** | **Number of Patients** | **Number of Events** | **Number of Patients** |
| *Any serious adverse event* |  | 10 | 7 (7%) | 15 | 12 (11.9%) | 25 | 19 (9.5%) |
| *Outcome* | Resolved | 10 | 7 (7%) | 15 | 12 (11.9%) | 25 | 19 (9.5%) |
| *Causality* | Unrelated to IMP & NIMP | 9 | 7 (7%) | 14 | 11 (10.9%) | 23 | 18 (9%) |
|  | Unrelated to IMP | 1 | 1 (1%) | 1 | 1 (1%) | 2 | 2 (1%) |
| *Expectedness* | Expected | 0 | 0 (0%) | 0 | 0 (%) | 0 | 0 (0%) |
|  | Unexpected | 10 | 7 (7%) | 15 | 12 (11.9%) | 25 | 19 (9.5%) |
| *Severity* | Mild | 5 | 5 (5%) | 5 | 4 (4%) | 10 | 9 (4.5%) |
|  | Moderate | 5 | 3 (3%) | 9 | 9 (8.9%) | 14 | 12 (6%) |
|  | Severe | 0 | 0 (0%) | 1 | 1 (1%) | 1 | 1 (0.5%) |

**Supplemental Table 4.**

Bleeding and Dyspnea events for safety population

|  | |  | **Ticagrelor**  **n=100** | | **Placebo**  **n=101** | | **Overall**  **n=201** | |
| --- | --- | --- | --- | --- | --- | --- | --- | --- |
|  | |  | **Number of Events** | **Number of Patients** | **Number of Events** | **Number of Patients** | **Number of Events** | **Number of Patients** |
| *Any bleeding event* | | | 88 | 64 (64%) | 14 | 12 (11.9%) | 102 | 76 (37.8%) |
| *PLATO classification* | Minimal | | 87 | 64 (64%) | 14 | 12 (11.9%) | 101 | 76 (37.8%) |
|  | Minor | | 1 | 1 (1%) | 0 | 0 (0%) | 1 | 1 (0.5%) |
|  | Major | | 0 | 0 (0%) | 0 | 0 (0%) | 0 | 0 (0%) |
|  | Major life threatening | | 0 | 0 (0%) | 0 | 0 (0%) | 0 | 0 (0%) |
| *Dyspnea* | At 1 year | | 27 | 24 (24%) | 8 | 8 (7.9%) | 35 | 32 (15.9%) |

**Supplemental Table 5.**

Post-hoc analysis of plasma high-sensitivity cardiac troponin I concentration (ng/L) in the intention to treat population who have measurement of troponin at 30 days

|  | Overall  (n=199) | Ticagrelor  (n=98) | Placebo  (n=101) |
| --- | --- | --- | --- |
| Coronary 18F-Fluoride Uptake | | | |
| N | 127 | 62 | 65 |
| Baseline | 3.8±2.9 | 4.2±2.9 | 3.5±2.9 |
| 30 days | 3.7±2.7 | 4.2±2.5 | 3.3±2.9 |
| Ratio of 30 days to baseline | 0.97±1.86 | 1.00±2.12 | 0.95±1.59 |
| No Coronary 18F-Fluoride Uptake | | | |
| N | 72 | 36 | 36 |
| Baseline | 2.4±2.5 | 2.5±2.7 | 2.4±2.4 |
| 30 days | 2.3±2.7 | 2.4±2.8 | 2.3±2.6 |
| Ratio of 30 days to baseline | 0.96±1.68 | 0.96±1.77 | 0.96±1.59 |

Geometric mean and geometric standard deviation, back transformed from log transformed values.

**Supplemental Table 6.**

Post-hoc analysis of plasma high-sensitivity cardiac troponin I concentration (ng/L) at 30 days for the intention to treat population

|  | **Adjusted Geometric Mean (GSE)** | | **Ratio of Geometric Means** |  |
| --- | --- | --- | --- | --- |
|  | **Ticagrelor** | **Placebo** | **(95% CI)** | **p-value** |
| Cardiac troponin I, ng/L  (*18F-fluoride activity*) | 3.9 (1.1) | 3.5 (1.1) | 1.12 (0.92 to 1.36) | 0.26 |
| Cardiac Troponin I, ng/L  (*No 18F-fluoride activity*) | 2.3 (1.1) | 2.3 (1.1) | 1.00 (0.78 to 1.29) | 0.98 |

*Estimates are back transformed estimates from analysis of log transformed values at 30 days adjusting for age, sex and log transformed baseline troponin. Ratio of geometric means is Ticagrelor divided by Placebo. GSE, geometric standard error.*

**Supplemental Table 7.**

Post-hoc analysis of plasma high-sensitivity cardiac troponin I concentration over 1 year for participants in intention to treat population

|  | **Adjusted Geometric Mean (GSE)** | | **Ratio of Geometric Means** |  |
| --- | --- | --- | --- | --- |
|  | **Ticagrelor** | **Placebo** | **(95% CI)** | **p-value** |
| AUC from 30 days to  1 year  (*18F-fluoride activity*) | 3.7 (1.1) | 4.3 (1.1) | 0.87 (0.64 to 1.17) | 0.35 |
| AUC from 30 days to  1 year  (*No 18F-fluoride activity*) | 2.4 (1.1) | 2.3 (1.1) | 1.04 (0.84 to 1.28) | 0.70 |

*Estimates are back transformed estimates from analysis of log transformed values area under curve from 30 days to 1 year adjusting for age, sex and log transformed baseline troponin. Ratio of geometric means is Ticagrelor divided by Placebo. AUC, area under curve, ng/L, GSE, geometric standard error.*

**Supplemental Figure 1.** Plasma high-sensitivity cardiac troponin I concentration (stratified population with troponin I >5ng/L at baseline) over 1 year.

Box-whisker plot of individual patient-level plasma high-sensitivity troponin I concentration (ng/L) in ticagrelor (blue) and placebo (red) groups at baseline, 1, 3, 6, 9 and 12 months (p=ns for all values). Median and interquartile range for each time point.


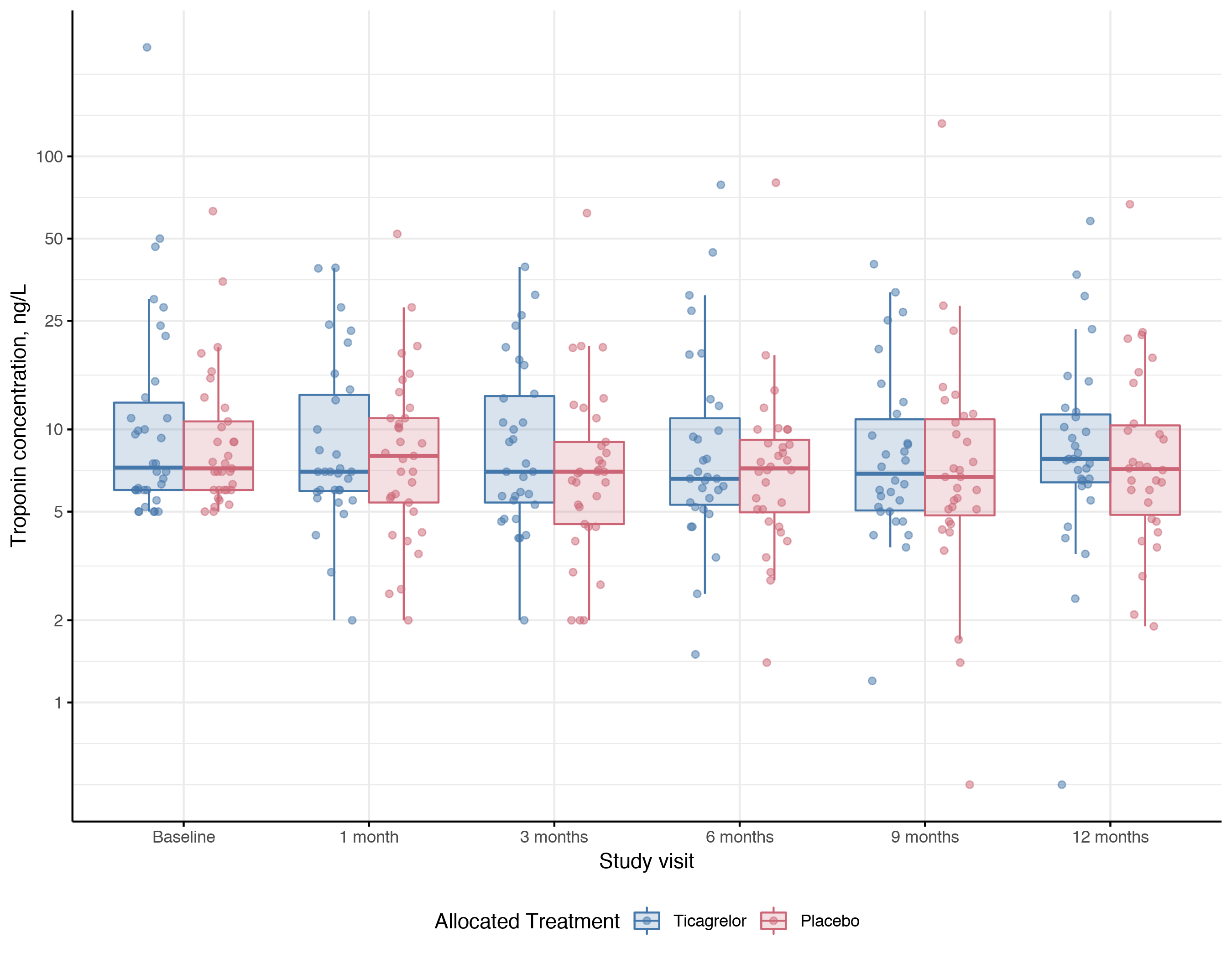

Supplement: Supplemental Tables 1–7 and Supplemental Figure 1 [file mmc1.docx]
